# Expression of Doublecortin, Glial Fibrillar Acidic Protein, and Vimentin in the Intact Subpallium and after Traumatic Injury to the Pallium in Juvenile Salmon, *Oncorhynchus masou*

**DOI:** 10.3390/ijms23031334

**Published:** 2022-01-25

**Authors:** Evgeniya V. Pushchina, Eva I. Zharikova, Anatoly A. Varaksin

**Affiliations:** A.V. Zhirmunsky National Scientific Center of Marine Biology, Far East Branch, Russian Academy of Sciences, 690041 Vladivostok, Russia; eva_1213@mail.ru (E.I.Z.); anvaraksin@mail.ru (A.A.V.)

**Keywords:** traumatic brain injury, adult neuronal stem cells, proliferation of cells, neuronal precursor cells, post-mitotic neuroblasts, subpallial part of telencephalon, masu salmon, GFAP, vimentin, doublecortin

## Abstract

Fetalization associated with a delay in development and the preservation of the features of the embryonic structure of the brain dominates the ontogeny of salmonids. The aim of the present study was to comparatively analyze the distribution of the glial-type aNSC markers such as vimentin and glial fibrillar acidic protein (GFAP) and the migratory neuronal precursors such as doublecortin in the telencephalon subpallium of juvenile masu salmon, *Oncorhynchus masou*, in normal conditions and at 1 week after an injury to the dorsal pallium. Immunohistochemical labeling of vimentin, GFAP, and doublecortin in the pallium of intact juvenile masu salmon revealed single cells with similar morphologies corresponding to a persistent pool of neuronal and/or glial progenitors. The study of the posttraumatic process showed the presence of intensely GFAP-labeled cells of the neuroepithelial type that form reactive neurogenic zones in all areas of the subpallial zone of juvenile masu salmon. A comparative analysis of the distribution of radial glia in the dorsal, ventral, and lateral zones of the subpallium showed a maximum concentration of cells in the dorsal part of subpallium (VD) and a minimum concentration in the lateral part of subpallium VL. An essential feature of posttraumatic immunolabeling in the masu salmon subpallium is the GFAP distribution patterns that are granular intracellular in the apical periventricular zone (PVZ) and fibrillar extracellular in the subventricular (SVZ) and parenchymal zones (PZ). In contrast to those in intact animals, most of the GFAP+ granules and constitutive neurogenic niches in injured fish were localized in the basal part of the PVZ. With the traumatic injury to the subpallium, the number of Vim+ cells in the lateral and ventral regions significantly increased. At 1 week post-injury, the total immunolabeling of vimentin cells in the PVZ was replaced by the granular pattern of Vim immunodistribution spreading from the PVZ to the SVZ and deeper parenchymal layers of the brain in all areas of the subpallium. A significant increase in the number of DC+ cells was observed also in all areas of the subpallium. The number of cells increased both in the PVZ and in the SVZ, as well as in the deeper PZ. Thus, at 1 week after the injury to the dorsal pallium, the number of DC, Vim, and GFAP expressing cells of the neuroepithelial type in the subpallium of juvenile masu salmon increased, and additionally GFAP+ radial glia appeared in VD, which was absent from intact animals.

## 1. Introduction

The telencephalon of teleost fishes, in contrast to the mammalian cerebral cortex, does not have a multilayer structure, but rather is formed by various cell clusters and extended cell populations and shows the ability to grow throughout life [1,2,3,4,5,6]. The ventral part of the telencephalon is formed by the subpallium, while the dorsal part by the pallium, which is subdivided into at least two rostral, two posterior, and two central divisions [7].

The pallial (dorsal) region of the telencephalon is formed through embryonic eversion during development [8], with the surfaces of the ventricles passing dorsally and laterally. A molecular marker expressed in the subpallium is factor Islet-1, found by immunocytochemistry in the ventral telencephalon of zebrafish [9]. The bodies of embryonic stem cells and radial glial cells in the pallium are located on the surface of the brain, surrounded by the ventricular space and tela choroidea, a remnant of the roof plate [6]. As a result of the eversion process, radial glial cells move towards the tela choroidea. Recent results of studies on perciforms have shown that, apart from the ventricular zone, there are very few proliferating cells in the dorsal pallium [6]. Nevertheless, earlier studies on juvenile salmon showed the presence of a limited number of BrdU-expressing cells [10] and a large number of PCNA-immunopositive cells in the pallium [11]. Thus, the proliferative potential of the telencephalon during active growth and the capability of increasing multifold in volume differ between various fish species [1,2,6,11]. These data apply more to the pallial regions of the brain than to the subpallium, which is much less studied.

Currently, there are several types of neurotransmitters, neurotrophic, and transcription factors that are known to be important for providing adaptive processes in the brain [12,13]. As factors controlling synaptic plasticity, most of them are also involved in various types of neural pathology, in particular, in brain injury or ischemic brain damage [14,15]. In all these cases, there occurs an inversion of the signaling function of neurotransmitters, whose insufficient or, vice versa, excessive activity becomes a cause of the disturbance of synaptic processes. The transformation of neural progenitors into mature cells of a specific mediator phenotype deserves special attention. The research data explain not only the fact of quantitative “multiplication” of new cells, but also the specifics of neurogenesis that cause neurons to form the desired mediator profile. According to developmental studies, the vertebrate telencephalon is divided into two major parts: the ventral region, or subpallium, and the dorsal region, or pallium. GABA-ergic neurons originate from the subpallium and can subsequently migrate to the pallium, as has recently been shown by Briscoe and Ragsdale [16] and was previously reported for zebrafish [17]. Thus, the inhibitory GABA-ergic expression dominates the subpallial telencephalic region, while the excitatory glutamatergic expression dominates the pallial dorsal telencephalic region. This general pattern inherent in mammals has also been confirmed in recent studies on the perciform fish *A. burtoni* [18].

Doublecortin (DC), a protein associated with microtubules and expressed exclusively by immature neurons, is used as a molecular marker of neuronal migration [19], which is a critical process in the nervous system development. The unique expression pattern of doublecortin allows it to be used as a marker of neurogenesis in adult animals. Mutations in this protein disrupt neuronal migration, leading to the development of pathological changes [20]. Currently, there are few works considering the use of DC as a marker of reparative neurogenesis in the brain of fish with central nervous system (CNS) trauma [10]. The use of molecular markers to verify neural stem cells (NSC), neuronal progenitors, and differentiated forms of neurons in the brain of adult mammals and other vertebrates yields somewhat contradictory results. Thus, GFAP and vimentin are considered as common markers of astrocytic glia in the vertebrate brain [6,21]. Nevertheless, typical astrocytes in the fish brain are not detected, while a limited population of cells that lack processes or have weakly expressed apical processes is verified, and widespread radial glial cells are also detected [22]. Subsequent studies have shown that radial glial cells are defined as non-neuronal cells with cell bodies located in the ventricular zone and bipolar processes extending to reach the pial and ventricular surfaces [23]. They are found in brains of all vertebrates, originate from neuroepithelial cells, and give rise to neurons and glia [3,23]. Ventricular and outer radial glial cells in the human brain development have also been characterized [12,24]. In the mammalian brain, most radial glial cells differentiate into astrocytes by the end of neuro- and gliogenesis, while in teleosts they retain their radial morphology [25]. Thus, radial glial cells are the dominant type of astroglia in the mature teleost brain. They support the proliferative capacity and, simultaneously, perform astroglial functions [3]. In the CNS of amniotes, postembryonic stem/progenitor cell populations usually have a glial phenotype: radial glial or astrocytic [26]. Neuroepithelial cells play an essential role in mammalian embryonic neurogenesis, while glial stem cells are the main source of neurons at the postembryonic stages of development [27]. In contrast to mammals, neuroepithelial-like (NE) stem/progenitor cells are present in the brain of fish throughout life [27,28,29].

However, the pattern of neurogenesis in adult animals based solely on glial cells, as inferred from these studies, needs further research, since it does not reflect the complexity of the biology and relationships of embryonic (eNSC) and adult (aNSC) vertebrates. Recent studies in mammals have shown that some ependymal cells play a potential role as “neural stem cells” [24]. Indeed, a subpopulation of ependymal cells has been reported to show stem cell activity in mice [27], and administration of vascular endothelial growth factor can reactivate resting ependymal cells in non-neurogenic areas of the adult brain [12].

The study of the neurogenic activity and proliferation of neuronal precursors in the zebrafish telencephalon, as a commonly applied model for neurogenetic research, allowed us to draw several preliminary conclusions. For example, a new zone of adult neurogenesis was identified in the dorsal telencephalon, which, apparently, has no analogues in the mouse brain [30]. Since this region in the zebrafish brain also contains components that reduce proliferative activity, it may represent a zone of NSC localization in the adult brain. It has now been established that neurogenesis in the olfactory bulb of fish is in many respects similar to that of mammals [30]. Progenitor cells in the fish telencephalon are found throughout ontogenesis, but in juvenile fish, the pattern of distribution of transcriptional and morphogenetic factors differs from that in the adult brain [6]. Thus, adult and postembryonic neurogeneses in the fish brain differ in the expression of molecular markers. Considered together, these data contribute to the understanding of not only phylogenetic, but also ontogenetic differences in neurogenesis between vertebrates.

Our earlier studies on the comparative distribution of BrdU-expressing cells, vimentin and GFAP as markers of astrocytic glia, and also doublecortin as a marker of migrating neuronal precursors in the pallium of juvenile salmon, *Oncorhynchus masou*, in normal conditions and after mechanical injury showed their complex dynamics and an ambiguous role in the post-traumatic period [10]. The aim of the present work was to study the dynamics of these molecular markers in the subpallium, addressing the following objectives:

*(i)*  Carry out a qualitative assessment of the distribution of GFAP-, vimentin-, and doublecortin-immunopositive elements in the proliferative zones of the subpallium of juvenile *O. masou* under conditions of constitutive neurogenesis;
*(ii)*  Assess the dynamics of the expression of the studied molecular markers in the post-traumatic period with mechanical trauma of the dorsal pallium;
*(iii)*  Conduct a comparative analysis and quantify the changes in the activity of the studied markers in the dorsal, ventral, and lateral zones of the subpallium.

## 2. Results

### 2.1. Doublecortin Immunohistochemical Labeling in the Intact Brain of Juvenile Masu Salmon

**Figure 1:**
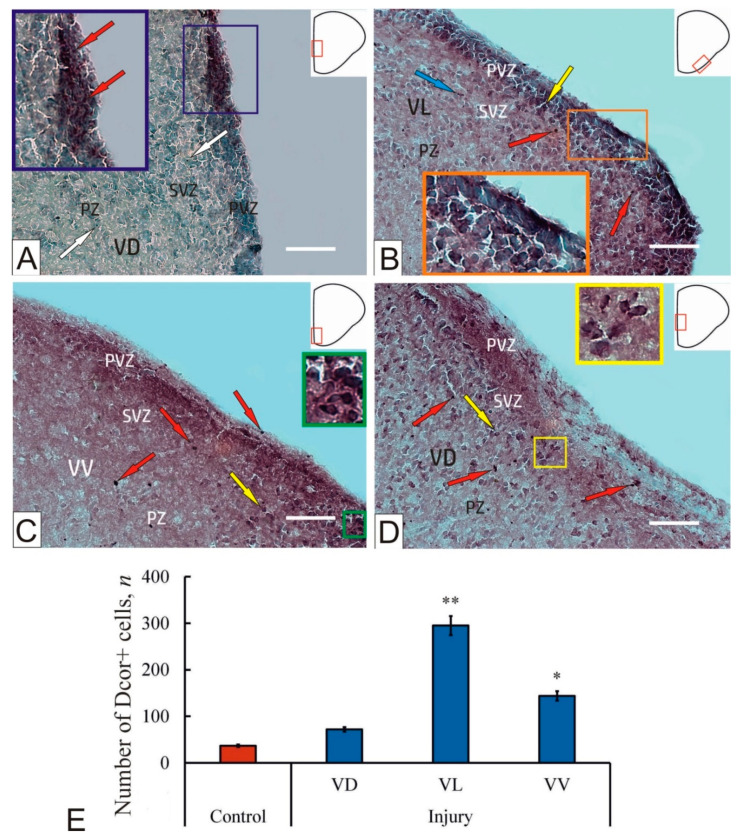
A representative image of doublecortin (DC) immunolabeling in the subpallial region of the intact telencephalon (**A**) of a juvenile *Oncorhynchus masou* and at 1 week after a traumatic injury (**B**–**D**) to the telencephalon: (**A**) localization of DC in intact subpallium, immunopositive cells (inset, red arrows), and granules (white arrows) in the parenchymal zone (PZ); (**B**) DC+ cells (inset) in lateral subpallial zone (VL), intensely DC-labeled cells (red arrow) and moderately DC-labeled cell (yellow arrow) in PVZ at 1 week post-injury, and DC+ granules (blue arrow); (**C**) post-traumatic expression of DC immunopositivity in ventral subpallial zone (VL), intensely labeled DC+ cells (red arrows) in PVZ and SVZ (an enlarged fragment is shown in the inset), and alternating with moderately-labeled cells (yellow arrow) in PZ; (**D**) patterns of DC-immunopositivity after injury in dorsal subpallium VD, intensely labeled cells (red arrows), moderately labeled cells (yellow arrows), and clusters of moderately labeled cells (inset); (**E**) the quantitative ratio of DC+ cells in intact animals (control group) and at 1 week post-injury (*n* = 5 in each group, * *p* ≤ 0.05; ** *p* ≤ 0.01; significant difference vs. control). Student–Newman–Keuls test. Immunohistochemical labeling of doublecortin in combination with methyl green staining. Scale bar: 100 μm.

When doublecortin was immunolabeled in intact animals in all areas of the subpallium, positive cells were detected mainly in the periventricular (PVZ) and subventricular (SVZ) zones; in the deeper parenchymal layers, immunopositive cells were isolated (Figure 1A). The cells were found in diffuse clusters of medium size (Figure 1A) or dense conglomerates (Figure 1A) consisting of several cells (Figure 1A). Moderately labeled elongated cells dominated the diffuse conglomerates (Figure 1A). In denser conglomerates, intensely labeled cells were located in the apical part of the PVZ, while less intensively labeled cells spread to the basal part (Figure 1A). Cell clusters of similar morphology were identified in the SVZ. As a result of microscopic analysis, several types of immunolabeled cells were identified at different stages of growth and differentiation, differing in size and topography (Supplementary Table S1). The first type of cells are small rounded cells with dimensions of 3.1 ± 0.6 × 2.4 ± 0.3 µm located in the immediate vicinity of larger elongated cells with dimensions of 5.3 ± 0.9 × 3.9 ± 0.5 µm. Dense aggregations of DC+ cells with varying degrees of differentiation, located directly in the PVZ and SVZ, corresponded to constitutive neurogenic niches.

### 2.2. Doublecortin Immunohistochemical Labeling in the Brain of Juvenile Masu Salmon after Injury

After injury, a significant increase in the number of doublecortin immunopositive cells was observed in all zones of the subpallium (Figure 1). The number of cells increased both in the PVZ and in the SVZ, as well as in the deeper parenchymal layers (PZ) (Figure 1B). The number of cells increased to a most significant value in the ventrolateral (VL) zone (*p* ≤ 0.01; Figure 1E). In addition to the number of cells in the subpallium of the masu salmon telencephalon, the pattern of immunolabeling of doublecortin changed and two types of positive cells (intensively and moderately labeled) appeared after mechanical injury (Figure 1B–D, Supplementary Table S1).

In the VL, a heterogeneous multi-row extensive layer of DC immunopositive cells in the PVZ was revealed, including cells of different intensities of DC immunolabeling, and immunonegative cells (Figure 1B). The pattern of cell immunolabeling, as a rule, allowed identification of a centrally located immunonegative nucleus, stained with methyl green, surrounded by a DC+ rim of cytoplasm (Figure 1B). In most cases, cytoplasmic immunolabeling was coarse-grained (Figure 1B). In some cases, the integrity of cell cytoplasm was disturbed, while immunopositive granules of subcellular size were visualized (Figure 1B). Nevertheless, the patterns of granular deposition of DC were sometimes visualized in the intercellular space, with the size of such granules being 0.5–1.5 µm. In the VL, two types of DC+ cells were identified (Supplementary Table S1). In the PVZ, in addition to horizontally oriented cells, there was an aggregation of intensely labeled.

DC+ cells, which represented a reactive neurogenic niche (RNN) that appeared in response to mechanical damage to the dorsal zone of the *O. masou* telencephalon. The sizes of doublecortin positive cells after damage also differed from those in the cells of the control (Supplementary Table S1). In particular, larger, moderately immunolabeled DC+ cells were identified in PVZ and SVZ. Intensively labeled large DC+ cells, absent in the control, were identified in the RNN (Figure 1C, Supplementary Table S1). The number of positive cells increased not only in the PVZ, but also in deeper parenchymal layers, with their diffuse distribution pattern also retained.

In the ventroventral zone (VV), single DC+ intensely labeled cells were detected in the apical part of the PVZ after injury (Figure 1C). A quantitative analysis showed a significant increase in the number of DC+ cells (*p* ≤ 0.05) compared to the control (Figure 1E). In the basal zone of the PVZ, DC+ cells of atypical morphology and a pseudo-unipolar form were sometimes identified, in which the expanded part was located in the basal part of the PVZ, and the narrower part spread to the apical zone of the PVZ (Figure 1C). In the same area, large immunonegative nuclei were visualized in cells with moderate DC labeling, and the pattern of immunolocalization was large-granular (Figure 1C). In the SVZ, single cells of this type were identified; in deeper parenchymal layers, single DC+ immunopositive cells were identified (Figure 1C).

In the ventrodorsal zone (VD), small, rounded or oval intensely labeled DC+ cells measuring 4–5 μm were detected in the post-traumatic period (Figure 1D; Supplementary Table S1). The number of such cells exceeded that in the control animals; nevertheless, compared to other zones in the VD, the smallest increase in the number of DC+ cells was revealed (Figure 1D.) Intensively labeled single DC+ cells of larger sizes were detected in the SVZ and PZ (Figure 1D). In general, compared to the control, immunopositivity was detected in larger, intensely, and moderately labeled cells in the VD after DC injury (Figure 1D, Supplementary Table S1).

### 2.3. GFAP Immunolabeling in the Intact Brain of Juvenile O. masou

**Figure 2:**
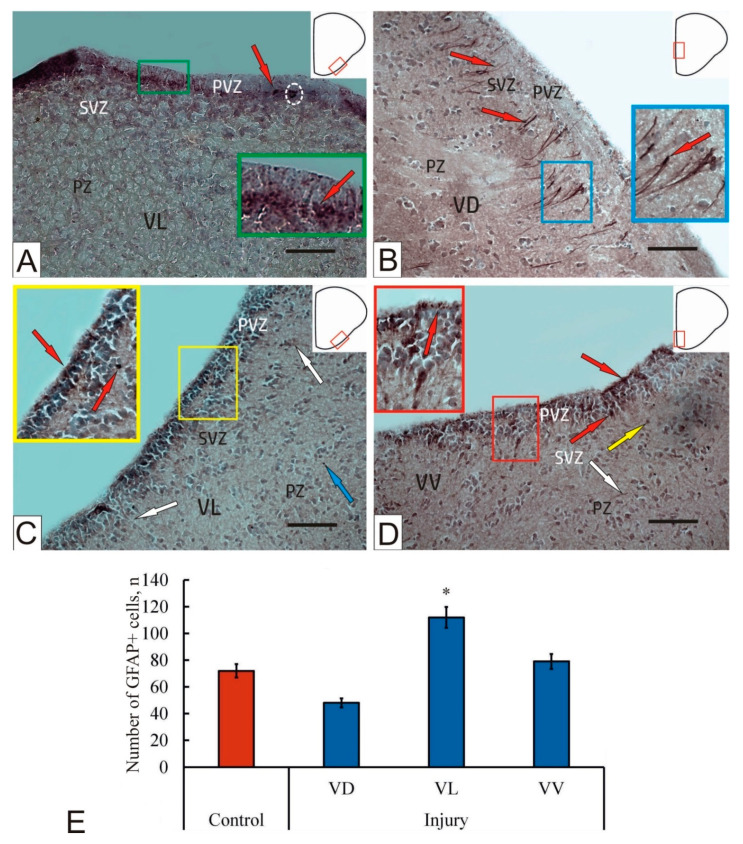
A representative image of GFAP-immunolabeling in the subpallial region of the intact telencephalon (**A**) of a juvenile *Oncorhynchus masou* and at 1 week after the traumatic injury (**B**–**D**) to the telencephalon: (**A**) localization of GFAP in intact lateral subpallium (VL), immunopositive cells (red arrows) and their clusters (white dashed oval) in PVZ (inset in the green rectangle); (**B**) GFAP+ cells conglomerates (red arrow) and clusters (inset in blue rectangle) in SVZ of VD at 1 week post-injury; (**C**) post-traumatic reorganization of GFAP immunopositivity in VL, GFAP+ heterogeneous complexes in PVZ and SVZ (an enlarged fragment is shown in the inset), alternating with immunonegative regions, radial glia fibers (white arrow), and GFAP+ granules (blue arrows); (**D**) heterogeneous GFAP+ cells after injury in the PVZ of VV (inset) and moderately labeled (yellow arrows), intensely labeled (red arrows), and radial glia (white arrows); (**E**) quantitative ratio of GFAP+ cells in intact animals (control group) and at 1 week post-injury (*n* = 5 in each group, * *p* ≤ 0.05; significant difference vs. control). Student–Newman–Keuls test. Immunohistochemical labeling of glial fibrillar acidic protein in combination with methyl green staining. Scale bar: 100 μm.

In the telencephalon of intact juvenile *O. masou*, small rounded or oval GFAP immunopositive cells were identified. In addition, numerous immunopositive granules less than 3 μm in size were clearly visualized. GFAP+ cells and granules formed constitutive clusters located along the basement membrane of the PVZ; some of the labeled elements were located closer to the apical part (Figure 2A). Larger immunopositive cells formed a dense conglomerate located in the SVZ (Figure 2A). Single immunopositive cells were found in the deeper layers of the parenchyma (Figure 2A, Supplementary Table S1).

### 2.4. GFAP Immunohistochemical Labeling in the Brain of Juvenile O. masou after Injury

As a result of traumatic injury in the subpallial VD, GFAP induction was detected in radial glial cells (Figure 2B, Supplementary Table S1). The posttraumatic expression of GFAP in radial glia (RG) cells was not typical for intact animals. Fibers of RG were most frequently recorded in the SVZ and to a lesser extent in the PZ (Figure 2B, Supplementary Table S1). As a rule, there were single fibers organized into small bundles, whose the distribution density varied in the dorsoventral direction. The intensity of GFAP immunolabeling varied from moderate to strong, with the thickness of RG fibers also varying. In some cases, cell migration patterns were identified along the labeled RG fibers (Figure 2B). In the PVZ, small granule-like deposits of GFAP and the surrounding clusters of immunonegative cells were identified (Figure 2B, Supplementary Table S1).

After the traumatic injury to in the lateral zone of the subpallium (VL), GFAP+ cells of the neuroepithelial type were detected in the PVZ, forming local reactive clusters containing 3–4 cells each (Figure 2C). In the SVZ, single GFAP+ cells with moderate immunopositivity were detected (Figure 2C), in the deeper parenchymal layers, a weakly labeled GFAP neuropil was detected that filled the intercellular space among immunonegative cells (Figure 2C, Supplementary Table S1). In the deeper layers of the PZ, numerous immunopositive cells with a granular pattern of immunolabeling were identified (Figure 2C).

In the ventral part of the subpallium (Vv), most of the GFAP+ elements were concentrated in the PVZ in the post-traumatic period (Figure 2D, Supplementary Table S1). In this area, reactive GFAP+ clusters were identified, including cells containing intensely labeled granules in the apical zone (Figure 2D). In the basal part of the PVZ, clusters of oval cells containing intensely labeled GFAP inclusions and, in some cases, GFAP+ processes were determined (Figure 2D). Such clusters alternated with areas that lacke RG and GFAP immunopositivity and contained dense conglomerates of immunonegative cells (Figure 2D). The combination of GFAP immunopositive regions with negative regions corresponded to the distribution of reactive neurogenic zones containing GFAP+ precursors in the PVZ. In other parts of the VD, zones of denser RG localization were identified, extending from the basal part of the PVZ to the SVZ (Figure 2D, Supplementary Table S1). Single regions of the PVZ contained an intensely labeled neuropil in the apical part, including individual GFAP+ granules in the extracellular and intracellular zones (Figure 2D). Separate moderately oval-shaped GFAP+ cells were identified in the SVZ (Figure 2D, Supplementary Table S1). In the PZ, aggregations of differentiated cells containing local granular regions of GFAP immunopositivity were determined, whose intensity varied from moderate to strong (Figure 2D). In the PZ, single multidirectional, moderately or intensely labeled fibers were identified (Figure 2D, Supplementary Table S1).

Thus, the study of the post-traumatic process in the acute period showed the presence of intensely labeled GFAP cells of the neuroepithelial type that form reactive neurogenic zones in all areas of the subpallial zone in juvenile *O. masou*. This post-traumatic stage was characterized in VD by induction of GFAP in RG cells, which is absent from intact animals. A comparative analysis of the distribution of RG cells in the dorsal, ventral, and lateral zones of the subpallium showed the maximum cell concentration in the VL and the minimum in the VD. An essential feature of posttraumatic immunolabeling in the subpallium of *O. masou* is GFAP distribution patterns that are granular intracellular in the apical PVZ and fibrillar extracellular in the SVZ and PZ. In contrast to those in intact animals, most GFAP+ granules and constitutive neurogenic niches were localized in the basal part of the PVZ.

The data of quantitative analysis showed a post-traumatic decrease in GFAP immunopositivity in the VD and a slight increase in the VV compared to the control (Figure 2E). A significant (*p* < 0.05) increase in the number of GFAP+ cells was detected only in the VD (Figure 2).

### 2.5. Vimentin Immunohistochemical Labeling in the Brain of Intact Juvenile O. masou

**Figure 3:**
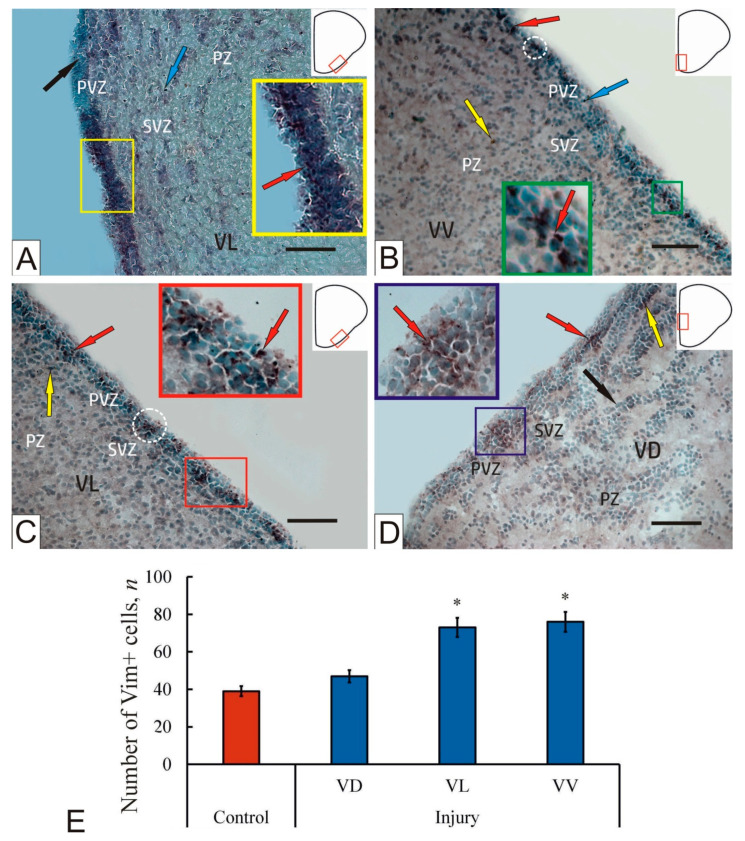
A representative image of Vim-immunolabeling in the subpallial region of the intact telencephalon (**A**) of a juvenile *Oncorhynchus masou* and at 1 week after the traumatic injury (**B**–**D**) to the telencephalon: (**A**) localization of Vim in intact subpallium, immunopositive cells (inset, red arrows) and granules (blue arrow) in the parenchymal zone (PZ), and Vim– cells (black arrow); (**B**) Vim+ cell conglomerates (inset) and clusters (white dashed oval) of intensely Vim-labeled cells (red arrow) and moderately Vim-labeled cell (yellow arrow) in PZ of VV at 1 week post-injury, and Vim+ granules (blue arrow); (**C**) post-traumatic reorganization of Vim-immunopositivity in VL, Vim+ heterogeneous conglomerates in PVZ and SVZ (an enlarged fragment is shown in the inset), alternating with immuno-negative regions, Vim+ cells clusters in PVZ (white dashed oval), intensely Vim-labeled cells (red arrows), and moderately Vim-labeled cells (yellow arrow); (**D**) weakly labeled Vim+ cells after injury in the PVZ of VD (inset), moderately labeled cells (yellow arrow), intensely labeled fibers (red arrows), and Vim– cell (black arrow); (**E**) the quantitative ratio of Vim+ cells in intact animals (control group) and at 1 week post-injury (*n* = 5 in each group, * *p* ≤ 0.05; significant difference vs. control). Student–Newman–Keuls test. Immunohistochemical labeling of vimentin in combination with methyl green staining. Scale bar: 100 μm.

In the subpallial region, vimentin immunolabeling in intact animals was detected in the PVZ. Intensively labeled intracellular granule-like inclusions of vimentin, forming extended areas alternating with zones of immunonegativity, were identified (Figure 3A, Supplementary Table S1). Along with the group patterns of vimentin distribution in the PVZ, single granules were identified (Figure 3A), and single Vim+ cells and granules were observed in the SVZ (Figure 3A). In the PZ, more extensive zones containing Vim+ cells and granules of parenchymal localization were identified, located within local clusters of immunonegative cells (Figure 3A). PZ regions without aggregations of migrating cells, as a rule, contained single Vim+ cells and granules (Figure 3A).

### 2.6. Vimentin Immunohistochemical Labeling in the Brain of Juvenile O. masou after Injury

In the ventral subpallial region in the post-traumatic period, local reactive aggregations of intensely labeled Vim+ cells were revealed in the basal part of the PVZ (Figure 3B, Supplementary Table S1). In the apical and medial parts of the PVZ, there were single or small clusters of Vim+ cells (Figure 3B). In the SVZ, single intensely and/or moderately labeled Vim+ granules, labeling subventricular cells, multidirectional weakly labeled neuropils, and RG fibers, were identified (Figure 3B, Supplementary Table S1). Separate Vim+ extra- and intracellular granules were found in the PZ (Figure 3B).

Clusters and single cells containing intensely labeled vimentin granules, organized in the form of reactive cell groups, were revealed in the VL in the PVZ (Figure 3C, Supplementary Table S1). Along with neuroepithelial, vimentin localization patterns in RG cells were revealed, which were absent from intact animals. In some areas of Vim, positive granules were localized in the basal part of the PVZ (Figure 3C). In some cases, reactive Vim+ cell clusters were identified in the basal part of the PVZ (Figure 3C). The density of distribution of immunonegative cells in such areas was increased compared to the areas of granular expression (Figure 3C). Moderately and weakly labeled fibers of RG were found in the area of the SVZ and deeper parenchymal layers (Figure 3C). Concentrations of weakly and moderately immunolabeled cells with cytoplasmic localization of vimentin were detected in the SVZ and PZ. Nevertheless, in the SVZ and PVZ, single intensely labeled granules of extracellular localization were identified (Figure 3C).

After traumatic injury in the VD, the PVZ contained clusters of moderately labeled Vim+ cells and tangentially oriented fibers forming local reactive clusters alternating with immunonegative regions (Figure 3D). In the PVZ, patterns of tangential cell migration were revealed in the post-traumatic period (Figure 3D). In some clusters, fragments of moderately labeled radial fibers in the PVZ and a multidirectional weakly labeled neuropil in the SVZ were identified. VD in the post-traumatic period is characterized by a pseudo-stratified spread of cell layers in the SVZ and PZ (Figure 3D). The space between the cell layers, as a rule, contained a multidirectional weakly labeled neuropil (Figure 3D). In some areas, intensely labeled vimentin granules were detected in the intercellular and intracellular space (Figure 3D).

Thus, as a result of traumatic injury in the subpallium, the number of Vim+ cells in the lateral and ventral regions significantly changed (*p* < 0.05) (Figure 3E). In the VD, the amount of Vim+ in the post-traumatic period slightly increased, but there was no significant difference (Figure 3E). In all areas of the subpallium, the total immunolabeling of vimentin cells in the PVZ was replaced by the granular pattern of Vim immunodistribution spreading from the PVZ to the SVZ and deeper parenchymal layers of the brain at one week after the traumatic injury.

## 3. Discussion

### 3.1. Expression of Doublecortin in the Telencephalon of Intact Juvenile O. masou and after Acute Injury

Doublecortin is a microtubulin-associated protein used as a molecular marker of neuroblasts required for the migration of immature neurons in the vertebrate brain [31]. Studies in mammals have shown the effective use of doublecortin as a marker of hippocampal neurogenesis in vertebrates [23,24,32]. Similar studies were also carried out for the telencephalon of birds: chicken and pigeon, in which proliferating PCNA- and doublecortin-positive cells were detected in the lumen of the lateral ventricles, labeling newly formed neurons in the telencephalon of adult animals [33]. Doublecortin is expressed by immature neurons, and its synthesis in neurons is associated with microtubules [19]. Neuronal migration is a critical process in the development of the nervous system. The unique expression pattern of doublecortin allows using it as a marker of neurogenesis in adult animals, while mutations in this protein disrupt the process of neuronal migration leading to pathological changes [20]. Despite the ability of doublecortin to modulate and stabilize microtubules for efficient migration, the mechanisms regulating this process are poorly understood. Disruptions in the interaction between doublecortin and microtubules destabilize the cytoskeletal organization, which leads to impaired cell migration [19]. Unlike other mitotic markers, the expression of doublecortin persists for a long time in young terminally differentiated neurons [34]. The expression of doublecortin plays a decisive role in the process of axonal growth and synaptogenesis in adult animals [35]. Doublecortin is expressed by newly created and migrating neurons in the intact subpallium of the *O. masou*, being localized both in cytoplasm and in nuclei of cells; its presence ensures the processes of intracellular motility. According to some data, the expression of doublecortin is retained in post-mitotic neuroblasts and may coincide with the expression of calretinin [36].

In our studies, intensely DC labeled cells and granules were found in the subpallial region of intact juvenile *O. masou* (Figure 1A). The study of the localization of doublecortin in the pallium of *O. masou* showed a low expression of the protein in cells of the primary neurogenic zones, with a predominance of the radial type of precursors in the dorsal zone of the pallium [10]. Compared to GFAP, the content of vimentin and doublecortin is almost twice as low in the PVZ of the subpallium, which indicates a relatively low content of post-mitotic neuroblasts in the PVZ. Nevertheless, in different areas of both intact subpallium and pallium, different quantitative content of DC+ granules was revealed. We assume that the expression of DC in the form of granules in the pallium and subpallium is necessary to ensure the neuronal plasticity. Studies on mammals have shown that DC is necessary for the formation of neurons in the adult brain [34]. In *Nothobranchius furzeri* [37] and *Astatotilapia burtoni* [6], DC-positive cells were detected in the telencephalon of adult animal, which obviously indicates similar DC functions in fish and mammals. Studies on mammals have shown that neurons formed in adulthood are capable of effective integration into existing neural networks, being involved in some aspects of behavioral plasticity [38]. Studies on birds have shown that DC expressing cells are characterized by different morphologies [33]. A high concentration of DC+ cells is characteristic of the pallial regions of the vertebrate brain; the caudal regions of the pallium are usually labeled more intensively than the rostral regions [33,34].

The results of studies on the subpallium of the juvenile *O. masou* brain differ from the data obtained on birds and other vertebrates; nevertheless, we associate the presence of DC+ cells in the subpallial region with the processes of neurogenesis and neuronal plasticity, which is consistent with the results of studies on *A. burtoni* [6], *N. furzeri* [37], and zebrafish [39]. The data obtained on the subpallium of juvenile *O. masou* also generally confirm the simultaneous process of radial migration of newly formed cells of the migration zone to the surrounding subpallial cell masses, which is consistent with previously obtained data on other fish species [37,39]. In studies on the cichlid *A. burtoni*, the growth of radial glia was analyzed using stem cell markers (nuclear antigen of proliferating cells PCNA and Sox2), and also doublecortin as a neurodifferentiation marker. As a result, these markers were found to be expressed on the ventricular surface in accordance with the stacking growth pattern [6]. In *A. burtoni*, doublecortin and Sox2-expressing cells were found in the deeper layers of the central pallium (Dc). These data suggest that radial glial cells give rise to migrating cells, delivering new neurons and glia to deeper regions of the telencephalon, which leads to the expansion of the central regions of Dc and displacement of the patterns of radial glia. Thus, the results of some studies [6] show that radial glial cells can adapt to morphological growth processes in the brain of adult fish and contribute to this growth.

As a result of damage to the pallium, the DC immunolabeling in the subpallium of juvenile *O. masou* was significantly altered. Currently, there are only a few studies considering the use of DC as a marker of reparative neurogenesis in mammalian brain [19]. For example, DC expression in the pallial part of the *O. masou* brain in the post-traumatic period was observed in the PVZ [10]. A characteristic feature, which was revealed by DC labeling, is a pronounced pattern of radial migration of cells from the PVZ to the deeper parenchymal layers of the brain. Such simultaneous migration is characteristic of neuroblasts formed in the post-traumatic period. The most pronounced increase in neuroblasts was in the lateral zone (*p* < 0.05) and slightly less pronounced in the ventral zone (*p* < 0.05), compared to the control animals (Figure 3 C,D). In the dorsal region, no significant increase in the number of DC+ cells was found. The appearance of intensely DC+ labeled cells in the brain parenchyma, in our opinion, indicates the activation of resident NSC pools as a result of the traumatic process, which is consistent with the data on mammals [19]. In the parenchymal zone of the pallium, a characteristic feature is the emergence of patterns of completed mitoses, which is a pair of closely spaced DC+ cells in the deep layers of the parenchyma, outside the proliferative zones [10]. Similar single structures were found in the VD zone of the *O. masou* subpallium (Figure 3B).

Small DC+ cells of parenchymal localization may correspond to the population of resident aNSCs, which are activated by a traumatic process. The presence of such cells corresponds to the localization of mesenchymal-type aNSC in mammals; such small cells show the highest proliferative activity, and their size, as a rule, does not exceed 5 µm [40]. The presence of a large number of such cell forms is characteristic of the pallium of juvenile *O. masou* [10] and is less pronounced in the subpallium. Thus, as a result of the traumatic process in the subpallial zone of the telencephalon, the aNSC pool is likely to be additionally activated, as well as the patterns of mass migration of newly formed DC+ neuroblasts migrating from neurogenic zones into the deep layers of the parenchyma.

### 3.2. Expression of GFAP in the Telencephalon of Intact Juvenile O. masou and after Injury

Currently, GFAP is considered as a marker of astrocytic glia in the vertebrate brain [21]. Nevertheless, typical astrocytes in the fish brain are often not detected; however, a population of cells without processes or with weakly expressed apical processes [22], as well as a population of RG cells, is verified [41]. Astroglial cells of the telencephalon in *A. burtoni* have a predominantly radial morphology [6], and their cell bodies form the surface of the ventricle. In fish studies, the term “radial glia” is used for glial cells located at the lumen of the ventricle, and the term “astroglia” is used in the case where cells of the astroglia family have been found in the brain parenchyma [6]. The shape and location of GFAP-immunopositive RG cells in *A. burtoni* clearly outline the transition from the ventricular surface of the dorsal pallium to the meningeal surface [6]. According to some date, GFAP labels the NSC population in the mammalian brain [20] and, thus, the issue of common application of NSC labeling in the brain of mammals and other vertebrates is not completely resolved.

Studies on the gray mullet *Chelon labrosus* have shown that GFAP expression in the brain changes throughout the lifetime, increasing with age [21]. In the early stages of development of *C. labrosus*, GFAP+ cell bodies, astrocyte endfeets, and tanycytes were detected, while typical expression in RG cells was usually observed at later stages of development. In studies of the subpallial zone of the telencephalon in juvenile *O. masou*, no GFAP+ cells with tanycyte-like morphology and/or typical process of astrocytes were found in the area of proliferative matrix zones. Previously, such cells were also not detected by GFAP-immunolabeling in the pallium [10]. In intact animals, single constitutive aggregations of uniformly labeled cells corresponding to constitutive neurogenic niches, as well as more extended zones with granular expression of GFAP of both intra- and extracellular localization, were identified in the basal part of the PVZ. We assume that the subpallium regions containing GFAP immunopositivity correspond to the regions with high neuronal plasticity containing adult tissue-specific neuronal progenitors, the presence of which determines a high neurogenic potential in the postembryonic development of juvenile *O. masou*. This is consistent with the data of studies on the mammalian CNS, for which the involvement of the population of astroglial cells, in particular, the ependymal radial glia and subependymal regions in the production of neurons, and also astrocytes and oligodendrocytes was established, which indicates the pluripotency of such cells of the corresponding aNSCs [28,42]. The presence of granule-like extracellular GFAP immunopositivity in the PVZ may indicate the neurotrophic involvement of GFAP in the process of constitutive neurogenesis. In mammals, RG is retained in the brain during the adult period [43]; however, no GFAP+ RG was found in the neurogenic niches of the *O. masou* activated as a result of injury. In studies on zebrafish, it has been shown that signs of reactive gliosis appear in the acute post-traumatic period [44], but then quickly cease and do not develop in such amount as those in mammals. For fish, the typical reactive astrogliosis that develops in the mammalian brain is not typical, since most areas surrounding the injury contain RG instead of astrocytes [45]. Taking into account the fact that part of the heterogeneous population of GFAP+ neuronal progenitor cells (NCP) express RG markers, while the other does not, it is highly probable that new neurons in the juvenile *O. masou* subpallium, as well as in the pallial zone [10], may arise from neuronal and non-neuronal cells predecessors. These assumptions are consistent with the results of studies on zebrafish, whose telencephalon retains the expression of neuronal genes: GFAP, GLAST, BLBP, and S100b [46].

In the post-traumatic period, the pattern of GFAP immunolocalization in the subpallium of juvenile *O. masou* significantly differed from the labeling pattern in intact animals. Along with neuroepithelial cells and patterns of granular immunolocalization in all studied subpallium zones, GFAP induction was detected in RG cells. Comparative studies and a quantitative analysis of the RG distribution in the subpallial areas showed the largest number of RG fibers in the VD (Figure 2E). This region was also characterized by the highest intensity of GFAP labeling in RG cells. GFAP+ elements appear as a result of the activation of glial-type resident aNSCs and their subsequent slow proliferation in response to traumatic effects. In juvenile *O. masou*, this is most pronounced in the VD, where the maximum density of distribution of RG fibers and a small number of granules were revealed. We assume that in the acute post-traumatic period, GFAP+ structures are reactive neurogenic niches containing glial-type aNSCs that arise in response to the injury. In the pallial region, similar changes, associated with the induction of RG, were found in the lateral region. In studies on zebrafish, RG aNSC and intermediate precursors have been identified [26]. According to [47], the source of eNSC in zebrafish is embryonic radial glia, which produces intermediate progenitor cells that are highly heterogeneous intermediate progenitor cells capable of active proliferation or dormancy [48] and expressing various molecular markers [49].

The revealed structural changes in the expression of GFAP in the subpallium of juvenile *O. masou* are similar with those in the pallium [10] and resemble the manifestations of acute gliosis in the mammalian brain. Nevertheless, the formation of a glial scar in the subpallium of the *O. masou* is not pronounced, which is consistent with the data for zebrafish [50]. According to Takedo and co-authors, GFAP+ processes of RG are involved in axonal restoration to a greater extent than in glial scar formation [51]. A detailed study of the cellular composition and patterns of subcellular and extracellular GFAP immunolocalization in the subpallium of juvenile *O. masou* indicates a multiple increase in plastic metabolism in the acute post-traumatic period [10]. A multiple increase in the GFAP+ NCP pool is another important consequence of the post-traumatic process; the presence of local reactive clusters containing intensely labeled cells indicates the proliferation of progenitor cells that increase the total number of cells involved in the reparative process. As a result of our studies, GFAP+ cells with neuroepithelial morphology were identified in the VV and VL, and VD with glial features after injury in the subpallium, thus indicating that the post-traumatic pattern of GFAP expression is associated with an increase in the expression of intermediate filament proteins.

### 3.3. Expression of Vimentin in the Telencephalon of Intact Juvenile O. masou and after Injury

Vimentin is a molecular marker of intermediate filaments that is often found in immature astrocytes and their differentiated forms [52]. Immunohistochemical studies of ependymocytes and radial glia in the brains of teleost fish showed the presence of vimentin [53]. An analysis of the amino acid sequence during sequencing of vimentin in teleost fish showed its homology with human vimentin [53,54]. The ratio of neuron- and gliospecific proteins labeling progenitor cell populations in the matrix zones of the telencephalon and cerebellum has species-specific features [26,55]. Vimentin-expressing cells were found in the telencephalon of various fish species: glial-type cells labeled with vimentin were identified in the pallium and subpallium of the zebrafish [53]. However, in the intact pallium of juvenile *O. masou*, most Vim+ cells had a neuroepithelial phenotype [10]. In early larvae of the mullet *C. labrosus*, weakly immunopositive radial glial cells were detected in the subpallium. In later larvae of *C. labrosus*, Vim expression is more pronounced, and reaches its maximum in large larvae [21]. In the pallium of juvenile *O. masou*, the expression of vimentin is relatively low [10]; in the subpallial region of intact juvenile *O. masou*, Vim+ cells in the SVZ and PZ were detected along with clusters of immunopositive cells in the PVZ. The data obtained are consistent with the results of immunolabeling of vimentin in the mullet subpallium, in which, however, most of Vim+ cells were identified around blood vessels in the ventral zone of the telencephalon [21].

After traumatic injury to the dorsal zone of the pallium in juvenile *O. masou*, an increase in the expression of vimentin in PVZ cells was observed in all areas of the subpallium, which is consistent with the data for zebrafish [30].

At one week post-injury, clusters of Vim+ cells and numerous Vim labeled cells, as well as single labeled cells in the brain parenchyma, were identified in the subpallium of juvenile *O. masou*. In contrast to the pallium, after injury in the subpallial region, aggregations of neuroepithelial cells in the PVZ were most characteristic. Typical patterns of RG localization in the subpallial region, in contrast to the pallium [10], were not identified in the post-traumatic period. In the parenchymal zone, on the contrary, moderate or intensely Vim labeled cells dominated. The results of quantitative analysis showed a significant increase in Vim+ cells in the VL and VV (*p* < 0.05) (Figure 3E). In the VD, no significant increase in the number of Vim+ cells was detected (Figure 3). Along with the increase in the number of immunopositive cells in the post-traumatic period, the number of Vim+ granules in the PVZ and SVZ increased, and the density of their distribution in the VD and VL increased significantly compared to the control (Figure 3A–C). An increase in vimentin expression at the intracellular level was often accompanied by an increase in Vim+ granules in dense reactive conglomerates of the neuroepithelial type in the lateral and ventral regions (Figure 3C,D). Our results on the subpallium of juvenile *O. masou* agree with the results of damage in the telencephalon of the zebrafish [50,56]. This fish was found to have the development of post-traumatic reactive gliosis, during which an increase in the expression of proteins of intermediate filaments, in particular vimentin, and also changes in the expression of some genes were observed [50,56]. As a result of damage to the telencephalon of zebrafish, the expression of vimentin, nestin, and calcium binding protein S100b in radial glial cells was increased [56]. Previous studies on the pallium showed similar structural changes in radial glial cells, as well as in posttraumatic GFAP labeling, after damage to the tegmentum of juvenile chum salmon *O. keta* [57]. Nevertheless, in the subpallial region of the telencephalon of juvenile *O. masou*, the post-traumatic expression of vimentin appeared mainly in neuroepithelial cells and their clusters corresponding to NSC/NPC of the embryonic type and was not detected in RG cells. The increase in the pool of Vim-ip cells in the lateral and ventral regions of the subpallium differed from the data obtained on the pallium [10]. The presence of local clusters of intensely labeled cells and granules of similar sizes and shapes indicated post-traumatic proliferation of neuroepithelial-type progenitor cells, which increased the total number of cells involved in the reparative process. Thus, the post-traumatic patterns of vimentin expression in the *O. masou* subpallium differ from the GFAP expression pattern; nevertheless, an increase in protein expression is observed in both cases. However, an important difference consists in the fact that GFAP expression was detected in both adult-type progenitors (RG) and neuroepithelial- type cells, whereas intracellular vimentin expression is characteristic mainly of neuroepithelial-type cells. Another important difference in post-traumatic vimentin expression is the extracellular granular distribution patterns that dominate in the PVZ.

## 4. Material and Methods

### 4.1. Experimental Animals

In the study, we used 30 one-year-old masu salmon (*Oncorhynchus masou*) with a body length of 11–13.5 cm and a body weight of 35–50 g. The animals were obtained in 2020 from the Ryazanovka experimental fish hatchery, where they had been kept in fresh water at a temperature of 14–15 °C, under 14/10-h light/dark cycle, and fed once a day. The concentration of dissolved oxygen in the water was 7–10 mg/dm^3^, which corresponds to normal saturation. All experimental manipulations with animals were carried out in accordance with the rules regulated by the charter of the A.V. Zhirmunsky National Scientific Center of Marine Biology, and the Ethical Commission regulating the humane treatment of experimental animals (permission no. 2 From December 10, 2021). The fish were anesthetized in a solution of tricaine methanesulfonate MS222 (Sigma, St. Louis, MO, USA) for 10–15 min.

After anesthesia, the intracranial cavity of the immobilized animal was perfused with a 4% paraformaldehyde solution prepared in 0.1 M phosphate buffer (pH 7.2) using a syringe. After prefixation, the brain was removed from the cranial cavity and fixed in a 4% paraformaldehyde solution for two hours at 4 °C. Then, the brain was kept in a 30% sucrose solution at 4 °C for two days (with a five-fold change of the solution). Serial frontal sections of masu salmon brain with a thickness of 50 μm were cut on a Cryo-star HM 560 MV freezing microtome (Germany), mounted on polylysine slides (Biovitrum, Russia), and dried.

### 4.2. Experimental Injury to the Telencephalon According to the Kishimoto’s Method

The animals were anesthetized in a cuvette with 0.1% tricaine methanesurfanate solution (MS-222, Aldrich, Sigma, USA) for 10 min at room temperature. Using a sterile needle (Carl Zeiss, Oberkochen, Germany), a mechanical injury was applied to the dorsolateral quadrant of the right hemisphere of the telencephalon to a depth of 1 mm. Immediately after the injury, the animals were released into the aquarium for recovery and further monitoring [58].

After the damage to the telencephalon region, video monitoring of changes in locomotor and behavioral activity in fish in the experimental group was carried out for 1 h. There were no significant changes in locomotor activity in animals with the telencephalon injury compared to the control group. A small hematoma measuring 1–2 mm was clearly visible in the area of injury.

### 4.3. Preparation of Material for Immunohistochemistry

After 1 week, the animals were withdrawn from the experiment and euthanized by the rapid decapitation method. The brain was perfused with a 4% paraformaldehyde solution prepared in 0.1 M of phosphate buffer (pH 7.2). After prefixation, the brain was removed from the cranial cavity and fixed in the same solution for 2 h at a temperature of 4 °C. Then it was washed in a 30% sucrose solution at 4 °C for two days, with a five-fold change in solution. Serial frontal sections of masu salmon brain with a thickness of 50 μm were cut on a freezing microtome (Cryo-star HM 560 MV, Walldorf, Germany).

### 4.4. Immunohistochemical Detection of Doublecortin, Vimentin, and Glial Fibrillar Acidic Protein

We used markers of astrocytic glia: glial fibrillar acidic protein (GFAP) and vimentin [21]. The protein doublecortin, associated with microtubules and expressed by immature neurons, was used as a marker of neuronal migration [19]. To study the expression of doublecortin, vimentin, and GFAP in the telencephalon of juvenile *O. masou*, immunoperoxidase labeling was used on frozen free-floating telencephalon slices. The brain sections were pre-incubated for 30 min at room temperature in PBS supplemented with 10% non-immune horse serum, 0.01% Tween 20 (Sigma, St. Louis, MO, USA), and 0.1% BSA (Sigma, St. Louis, MO, USA). We used monoclonal mouse antibodies against GFAP (GF5 Catalog No. ab10062), vimentin (3B4 Catalog No. ab28028) from Abcam (Cambridge CB2 0AX, UK), and doublecortin (CO613 Catalog No. sc-271390; Santa Cruz Biotechnology, Santa Cruz, CA, USA), at a dilution of 1:300. Protein activity was assessed at 7 days after the traumatic injury to the telencephalon region. Transversal sections with a thickness of 50 µm were incubated in situ at 4 °C for 48 h.

For visualization of immunohistochemical (IHC) labeling, a standard ABC complex Vectastain Elite ABC kit (Vector Laboratories, Burlingame, CA, USA) was used in accordance with the manufacturer’s recommendations. To identify the reaction products, a red substrate (VIP Substrate Kit, Vector Labs, Burlingame, CA, USA) was used. The preparations were mounted on polylysine-coated microscope slides (BioVitrum, Russia, St. Petersburg) and left to dry completely. Then, to identify immunonegative cells, the preparations were counterstained with 0.1% methyl green solution (Bioenno, Lifescience, Santa Ana, CA, USA, Cat # 003027). Color development was monitored under a microscope, washed with distilled water for 10 s, then differentiated for 1–2 min in a 70% alcohol solution, and then for 10 s in 96% ethanol. The brain sections were dehydrated, according to a standard technique, in two changes of xylene, 15 min each, and then placed in Bio-optica medium (Italy) under cover slips.

To assess the specificity of immunohistochemical reaction, a negative control method was used. Instead of primary antibodies, brain slices were incubated with a 1% solution of non-immune horse serum for 1 day and processed as slices with primary antibodies. In all control experiments, there was no immunopositive reaction.

### 4.5. Microscopy

For visualization and morphological analysis, a motorized inverted microscope with a fluorescent module and an attachment for improved contrast enhancement was used when working with Axiovert 200 M luminescence with an ApoTome module (Carl Zeiss, Germany). Micrographs of brain sections were taken, and analysis of the material performed using the Axio Vision software (Carl Zeiss, Oberkochen, Germany). Measurements were carried out using objectives with a magnification of 10×, 20×, 40×, and 63× in several randomly selected fields of view for each study area. The number of immunolabeled cells in the field of view was counted at a magnification of 200×. Morphometric analysis of the parameters of cell bodies (measurement of large and small diameters of the soma of neurons) was carried out using the software of the Axiovert 200 M microscope. Micrographs of the preparations were taken using an Axiovert 200 digital camera. The material was processed using the Axioimager software and the graphics editor Corel Photo-Paint 15.

### 4.6. Densitometry

The optical density (OD) of IHC labeling products in the bodies of neurons and immunopositive granules was measured using the Axiovert 200 M microscope software. Then, using the Axio Vision software, a standard determination of optical density was performed for intensively and moderately labeled and immunonegative cells. Optical density (OD) in immunopositive cells was categorized by the following scale: high (180–130 units of optical density (UOD), corresponding to +++), moderate (130–80 UOD, corresponding to ++), weak (80–40 UOD, corresponding to +), and low (less than 40 UOD, corresponding to –). The initial OD value was measured on the control mounts. For obtaining the actual value in relative units of optical density, the average value of optical density for each type of cells was subtracted from the maximum value of optical density for immunonegative cells (background). Based on the densitometric analysis data, various levels of GFAP, Vim, and DC activity in cells were determined. These data, along with the dimensional characteristics, were used for identification of the cell type cells on the basis of the previously developed classification of cells in the pallial zone of the *O. masou* telencephalon [10] formed during the period of constitutive and reparative neurogenesis.

### 4.7. Statistical Analysis

Quantitative processing of the material was carried out using the Descriptive Statistics programs in Microsoft Excel 2010 and Statistica 12. The density of distribution and size characteristics of cells were estimated using the methods of variation statistics. To quantify the results, mean values and standard deviation (M ± SD) were calculated. All group measurements were compared using the Student–Newman–Keuls test. Values at *p* ≤ 0.05 were considered statistically significant.

## 5. Conclusions

The studied molecular markers—doublecortin, GFAP, and vimentin—made it possible to identify few or single immunopositive cells in the subpallium of intact juvenile *O. masou*. A subsequent analysis of these cells, identified for the area of the matrix zones of the subpallium, showed that these are basically small, rounded, and oval cells of the same type as those detected in all variants of immunolabeling, which allows us to consider them as a persistent pool of neuronal and/or glial progenitors present in the subpallial part of the matrix zone in juvenile *O. masou*. Immunopositive DC, GFAP, and Vim granules of subcellular localization of various sizes and distribution densities were consistently detected. Such granules were found both in the cytoplasm of the cells in the matrix zones and as elements of the extracellular matrix.

In the subpallial area of the *O. masou* brain, intense DC labeling of cells in the periventricular layers of the brain was observed after injury. A characteristic feature of DC labeling post-injury is the pattern of cell migration from the dorsal neurogenic zone of the subpallium into the depth of the brain parenchyma. This confirms the simultaneous process of radial migration of numerous neuroblasts newly formed as a result of the traumatic process. The appearance of intensely DC-labeled cells in the brain parenchyma indicates the activation of the pools of resident NSCs as a result of the traumatic process. In the PZ of the subpallium, the appearance of closely spaced pairs of intensely DC-labeled cells located outside the proliferative zones is characteristic after injury. In the VD, in contrast to the VL and VV, highly immunogenic cells were identified, both in the subpallial parenchyma and in the area of the PVZ. The most numerous DC-labeled migrating cells with moderate DC-immunoreactivity were identified in VD. Thus, as a result of the traumatic process, an additional pool of highly active aNSCs as well as patterns of mass migration of newly formed DC+ neuroblasts, migrating from neurogenic zones into the deep layers of the parenchyma, are formed in the *O. masou* subpallium.

The pattern of GFAP immunolabeling in the subpallium after the damage to the pallium of juvenile *O. masou* was significantly different from the GFAP labeling in intact animals. In the VD, fibers of radial glia and single small, intensely GFAP-labeled cells in the parenchyma appear. All of these GFAP+ elements appear de novo as a result of the activation of resident glial-type aNSCs and their subsequent slow proliferation in response to injury. This is most pronounced in juvenile *O. masou* in the VL, where heterogeneous clusters were found, including both GFAP-positive and GFAP-negative cells, as well as GFAP-positive granules. We suggest that these GFAP-immunopositive structures represent reactive neurogenic niches containing glial-type aNSCs that arise in response to injury. A detailed study of the cellular composition and patterns of extracellular immunolocalization of vimentin in the subpallium of juvenile *O. masou* after injury has shown a multiple increase in the processes of plastic metabolism. Another important observation is the multifold increase in the Vim+ NPC pool formed as a result of injury. Thus, the post-traumatic vimentin expression patterns are largely reminiscent of GFAP expression, which indicates a similar increase in the expression of intermediate filament proteins upon damage to pallium in juvenile *O. masou*. As a result of our studies, Vim+ cells with neuroepithelial morphology were identified in the VD and VV, and cells with glial morphology in the VL after injury. This extends the previously obtained data on zebrafish and medaka and shows the species-specific distribution of aNSC in the fish subpallium.

The obtained data raise various questions for further studies of adult neurogenesis in the pacific salmon brain as follows. How does the ratio of neuroepithelial and glial progenitors change, and does it change at all, during the further development of the brain of juvenile *O. masou*? How does the growth rate in the subpallium change at the later stages of postembryonic ontogenesis of the *O. masou* brain, because the brain size of an adult *O. masou* is multifold larger than that of juveniles? From what types of predecessors is the structure of various telencephalic subregions formed during the further development in *O. masou*. Answers to these questions can significantly advance the understanding of the neurogenesis in salmonids as convenient models for brain research.

## Supplementary Materials

The following supporting information can be downloaded at: https://www.mdpi.com/article/10.3390/ijms23031334/s1.

## Abbreviations

aNSCs: adult neural stem cells; DC+, doublecortin positive cells; VD, dorsal part of subpallium; VL, lateral part of subpallium; VV, ventral part of subpallium; IHC, immunohistochemistry; GFAP+, glial fibrillar acid protein positive cells; NPCs, neuronal precursor cells; NSCs, neural stem cells; PVZ, periventricular zone; PZ, parenchymal zone; SVZ, subventricular zone; Vim+, vimentin positive cells.
