# Supplementary material for: Expression of Doublecortin, Glial Fibrillar Acidic Protein, and Vimentin in the Intact Subpallium and after Traumatic Injury to the Pallium in Juvenile Salmon, Oncorhynchus masou
Source: Int J Mol Sci. 2022 Jan 25;23(3):1334. doi: 10.3390/ijms23031334 (PMC8836249; doi:10.3390/ijms23031334)
Supplement: Supplementary file 1 [file ijms-23-01334-s001.zip › ijms-1547956-supplementary-done.pdf]

International Journal of
Molecular Sciences

MDPI

Table S1. Morphometric parameters of doublecortin+, GFAP+ and vimentin+ cells (M ± SD) in the subpallial dorsal (VD), lateral (VL) and ventral (VV) zones of the intact telencephalon of juvenile masu salmon (*Oncorhynchus masou*) and after injury

**Intact animals**		**Damaged telencephalon**					
		**VD**		**VL**		**VV**	
Cell size, μm/ Localization	Optical density, UOD	Cell size, μm/ Localization	Optical density, UOD	Cell size, μm/ Localization	Optical density, UOD	Cell size, μm/ Localization	Optical density, UOD
**Doublecortin**							
11.4±1.0/7.2±1.3 (PZ)<br>9.0±0.4/5.9±0.4 (SVZ)<br>7.4±0.2/4.2±1.0 (PVZ)<br>6.1±0.5/3.7±0.7 (PZ)	+++<br>+++<br>+++<br>+++	9.0±0.5/6.0±0.3 (SVZ)<br>8.2±0.3/6.7±0.5 (PZ)<br>6.2±0.3/5.1±0.2 (SVZ)<br>4.9±0.2/3.0±0.2 (PVZ)	+++<br>+++<br>+++<br>+++	8.6±0.7/6.1±0.7 (PZ)<br>7.5±0.3/4.5±0.5 (SVZ)<br>6.7±0.4/4.9±0.6 (PVZ)<br>5.8±0.4/4.4±0.4 (PVZ)	+++<br>+++<br>+++<br>+++	8.6±0.5/6.1±0.5 (PVZ)<br>7.3±0.3/5.3±0.7 (PVZ)<br>5.9±0.5/4.6±0.5 (PVZ)	+++<br>+++<br>+++
3.1±0.6/2.4±0.3 (PVZ)<br>5.3±0.9/3.9±0.5 (PVZ)<br>7.4±0.2/4.2±1.0 (SVZ)	++<br>++<br>+	11.2±0.6/8.1±1.0 (PVZ)<br>9.3±0.5/6.9±0.8 (SVZ)	++<br>++	9.3±0.9/6.0±1.1 (PVZ)<br>7.8±0.1/5.7±0.6 (SVZ)<br>7.0±0.2/5.0±0.4 (PVZ)<br>6.1±0.5/5.1±0.6 (PVZ)	++<br>++<br>++<br>++	8.5±1.0/5.6±0.6 (PVZ)<br>7.9±0.3/6.2±0.5 (PVZ)<br>7.1±0.3/4.5±0.4 (PVZ)<br>7.0±0.3/5.5±0.6 (PVZ)<br>5.9±0.5/4.4±0.4 (PVZ)	++<br>++<br>++<br>++<br>++
**GFAP**							
9.7±1.0/6.2±1.3 (SVZ)<br>7.4±0.4/4.8±0.5 (PVZ)	+++<br>+++	10.8±0.3/8.1±0.7 (PZ)<br>9.8±0.2/6.4±0.2 * (PVZ)<br>6.0±0.6/3.2±0.5 (PVZ)	+++<br>+++<br>+++<br>+++	9.6±0.4/7.2±0.7 (PZ)<br>7.5±0.8/5.5±0.3 (PVZ)<br>6.2±0.4/4.5±0.7 (SVZ)	+++<br>+++<br>+++<br>+++	8.9±0.5/5.7±0.5* (PVZ)<br>7.8±0.2/6.6±0.3 (PVZ)<br>7.4±0.5/5.0±0.3(PZ)	+++<br>+++<br>+++

		4.0±0.2/2.4±0.3 (PVZ)		5.3±0.4/3.3±0.4 (PVZ)			
6.5±0.3/4.6±0.6 (SVZ) 5.0±0.5/3.8±0.6 (PVZ) 11.5±0.5/7.7±1.5 (PVZ)	++ ++ +	11.4±0.8/6.4±1.3 (PVZ) 8.6±0.5/7.2±0.5 * (PVZ) 8.4±1.0/5.3±0.4 (PZ)	++ ++ +	10.2±0.6/8.4±0.7 (SVZ) 8.8±0.3/6.7±0.7 * (PVZ) 7.1±0.5/5.0±0.4 (PVZ)	++ + ++	10.2±0.6/7.3±0.7 (SVZ) 8.4±0.2/6.1±0.2* (PVZ) 8.3±0.5/5.1±0.6 (SVZ)	++ ++ +
**Vimentin**							
9.7±0.5/6.8±0.4 (PVZ) 8.3±0.3/5.6±0.4 (PVZ)	+++ +++ ++	8.0±0.7/5.6±0.6 (PZ) 6.6±0.4/5.3±0.7 (PVZ) 6.3±0.5/3.9±0.5 (PVZ)	+++ +++ +++	10.6±0.3/7.3±0.8 (PZ) 8.6±0.2/5.3±0.2 (PVZ) 7.0±0.5/5.3±0.9 (PVZ)	+++ +++ +++	6.4±0.3/4.2±0.4 (PZ) 5.6±0.2/4.0±0.6 (SVZ)	+++ +++
7.3±0.3/5.3±0.7 (SVZ) 6.3±0.5/4.6±0.8 (PVZ)	++ +	9.3±0.6/6.5±0.6 (PVZ) 7.9±0.2/5.7±0.8 (SVZ) 6.9±0.5/5.2±0.9 (SVZ)	++ ++ ++	7.6±0.7/5.0±0.5 (PVZ) 5.9±0.5/4.3±0.3 (SVZ)	++ ++ ++	10.0±0.8/6.7±0.8 (SVZ) 8.1±0.5/4.9±0.5 (SVZ) 7.9±0.7/6.6±0.6 (PVZ) 5.9±0.7/4.5±0.6	++ ++ + ++

Optical density (OD) in immunopositive cells was categorized by the following scale: high (180–130 units of optical density (UOD), corresponding to +++), moderate (130–80 UOD, corresponding to ++), weak (80–40 UOD, corresponding to +), and low (less than 40 UOD, corresponding to –); the initial OD value was measured on the control mounts. The greater and lesser diameters of the cell body are shown as values divided by slash. Cells were morphologically classified on the basis of a previously developed scheme [10]. Asterisk (*) indicates radial glia that appears in the intact *O. masou* telencephalon and/or after injury.
